# Are mother-baby units the standard of care for the treatment of postnatal mental health disorders

**DOI:** 10.1192/j.eurpsy.2025.574

**Published:** 2025-08-26

**Authors:** C. P. Desport, R. F. Dionísio

**Affiliations:** 1 Hospital de Magalhães Lemos, Unidade Local de Saúde de Santo António, Porto, Portugal

## Abstract

**Introduction:**

The postpartum is a high-risk period for onset and recurrence of maternal mental health disorders. If left untreated these disorders can have important consequences on mother, baby and family. When the disorder is severe or there are no conditions to manage it in the community the mother may need to be hospitalized and the gold-standard are the mother-baby units (MBU) in which mothers are co-admitted with their infant.

**Objectives:**

We aim to explore this model of care (MBU), the pros and cons and how it has been done worldwide, so you can better understand how they work and improve the care we provide to our patients.

**Methods:**

To this purpose we conducted a literature review by searching and analyzing papers on mother-baby units. The search was on PubMed and Google Scholar, with the following key-words: “mother-baby unit”, “maternal mental health”, “postpartum mental health” and “postnatal psychiatry”.

**Results:**

The MBU are considered the best practice by the National Institute for Health and Care Excellence (NICE) and their guidelines for Antenatal and Postnatal Mental Health recommend MBUs be staffed by specialist perinatal mental health staff. The postnatal period is a critical time for mother and baby binding and there is scientific evidence that separating the mother and infant during the first year could impact negatively on the developing attachment relationship and be a detrimental and traumatic experience for both. Also, by committing both mother and baby, we have an opportunity to watch and guide the mother to baby care. The United Kingdom pioneered in MBUs and in 1958 opened the first separate MBU and then France opened theirs in 1979 and Belgium in 1990.

**Conclusions:**

To summarize, the dyad admission, allows for the mother to receive adequate treatment for her psychiatric disorder, whilst also receiving support in developing parenting skills. In the last years many countries worldwide have adopted this as standard of care, with excellent outcomes for both mother and baby.

**Disclosure of Interest:**

None Declared

